# Analysis of Olive (*Olea Europaea* L.) Genetic Resources in Relation to the Content of Vitamin E in Virgin Olive Oil

**DOI:** 10.3390/antiox8080242

**Published:** 2019-07-25

**Authors:** Ana G. Pérez, Lorenzo León, Mar Pascual, Raúl de la Rosa, Angjelina Belaj, Carlos Sanz

**Affiliations:** 1 Department of Biochemistry and Molecular Biology of Plant Products, Instituto de la Grasa, CSIC, 41013 Seville, Spain; 2 IFAPA, Centro Alameda del Obispo, 14004 Cordoba, Spain

**Keywords:** *Olea europaea* L., virgin olive oil, vitamin E, tocopherols, variability, crop year, ripeness, quality, breeding

## Abstract

Virgin olive oil (VOO) is the main source of lipids in the Mediterranean diet and one of the main contributors to its proven protection against diseases associated with chronic inflammation states. This oil is rich in antioxidant compounds such as tocopherols, which together constitute the vitamin E stock of the oil. The purpose of the present work was to conduct a study on the diversity of the contents of vitamin E in the olive species (*Olea europaea* L.), and to know how the season climatic conditions and the degree of fruit ripening stage influences the final content of this vitamin in VOO. Data showed that the content of vitamin E in VOO is highly dependent on the olive cultivar, displaying a wide variability (89–1410 mg tocopherol/kg oil) in the olive species, and that is also dependent, to a lesser extent, on the crop year climate and the stage of fruit ripening. In addition, the suitability of cultivar crosses for breeding programs to obtain new cultivars with improved vitamin E content in VOO has been assessed. Our findings demonstrated that a single cross of olive cultivars may provide sufficient variability to be used in the selection of new cultivars.

## 1. Introduction

Much of the recognized nutritional benefits of the Mediterranean diet are provided by its main source of lipids, virgin olive oil (VOO). The regular consumption of VOO could provide sufficient amount of antioxidant compounds to attenuate the inflammatory response of the body tissues and thus reduce the risk of suffering diseases associated with chronic inflammation states [1,2,3]. VOO is consumed directly as the juice of the olive fruit and therefore contains many of the secondary metabolites that are stored in the fruit and constitute an extraordinarily rich food in bioactive compounds. Among them, the content of phenolic compounds stands out, given that they exhibit potent antioxidant properties. VOO phenolics mainly comprise phenolic compounds with secoiridoid structures [4] and tocopherols. The latter constitute the vitamin E content of the oil. In this regard, it is also important to highlight the well-known synergistic effect that secoiridoid phenols and vitamin E have in relation to the antioxidant properties of VOO [5]. The role of vitamin E is based on its key function as antioxidant agent that limits lipid peroxidation in cell membranes and scavenges reactive oxygen species, protecting tissues from consequent oxidative damage [6,7]. Beneficial effects of supra-nutritional doses of vitamin E have been reported for cardiovascular diseases, cancer, chronic inflammation, Alzheimer’s disease, and Parkinson’s disease [8]. Thus, the European Food Safety Agency allowed a nutritional claim [9] declaring “a cause and effect relationship has been established between the dietary intake of vitamin E and protection of DNA, protein and lipids from oxidative damage.”

**Figure 1:**
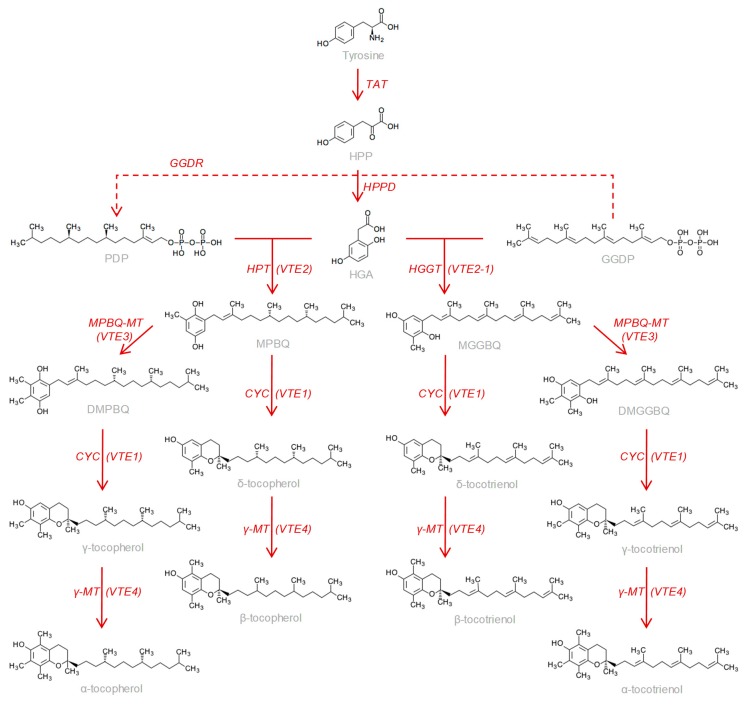
Biosynthetic pathway for tocopherols and tocotrienols in plants. Abbreviated intermediate metabolites are: hydroxyphenylpyruvate (HPP); phytyl diphosphate (PDP); homogentisate (HGA); geranylgeranyl diphosphate (GGDP); 2-methyl-6-phythylbenzoquinol (MPBQ); 2,3-dimethyl-6-phythylbenzoquinol (DMBQ); 2-methyl-6-geranylgeranylbenzoquinol (MGGBQ); 2,3-dimethyl-6-geranylgeranylbenzoquinol (DMGGBQ). The genes are the following: tyrosine aminotransferase (TAT); geranylgeranyl-diphosphate reductase (GGDR); 4-hydroxyphenylpyruvate dioxygenase (HPPD); homogentisate phytyltransferase (HPT/VTE2); homogentisate geranylgeranyltransferase (HGGT/VTE2-1); 2,3-dimethyl-6-phythylbenzoquinol methyltransferase (MPBQ-MT/VTE3); tocopherol cyclase (VTE1); γ-tocopherol methyltransferase (VTE4).

The term ‘vitamin E’ covers eight different vitamers with a tocochromanol structure that are produced solely by plants, four tocopherols and four tocotrienols which, depending on the number and position of methyl groups in the ring, are identified as α, β, γ, and δ forms. Tocochromanols are synthesized from the condensation of homogentisate (HGA), synthesized from tyrosine, and phytyl (PDP) or geranylgeranyl diphosphate (GGDP) by the action of homogentisate phytyltransferase (HPT/VTE2) to form 2-methyl-6-phytyl-1,4-benzoquinol (MPBQ) or homogentisate geranylgeranyltransferase (HGGT/VTE2.1) to form 2-methyl-6-geranylgeranyl-1,4-benzoquinol (MGGBQ) (Figure 1). These intermediates may be cycled by tocopherol cyclase (CYC/VT1) before or after methylation by MPBQ methyltransferase (MPBQ-MT/ VTE3) to form δ- and γ-tocochromanols, respectively. Finally, the methylation of δ- and γ- tocochromanols by γ-tocopherol methyltransferase (γ-MT/ VTE4) results in the production of β- and α-tocochromanols, respectively.

α-Tocopherol is the most common form and presents greater biological activity because it is the only form incorporated into very low-density lipoproteins (VLDL) by means of a specific liver protein [8]. Few olive cultivars have been characterized in relation to their vitamin E content. They all seem to have in common that α-tocopherol is the most abundant vitamer in VOO, which also contains, to a much lesser extent, β and γ-tocopherol [10,11,12,13]. However, the content of a fourth form of tocopherol, δ-tocopherol, in addition to the four tocotrienols forms (α, β, γ, and δ) has been recently quantified in the oils from five different Greek olive cultivars [13,14]. Unlike the phenolic compounds with secoiridoid structures of VOO, whose biosynthesis begins with the loss of cell integrity during the grinding of the fruit that allows to put in contact primarily phenolic glycosides and glycosidic enzyme activities, the content of vitamin E in VOO seems to suffer little variation by the conditions of the extraction process [15,16,17], genetic and agronomic factors being the main factors that determine the final concentration of vitamin E in the oil.

In recent times, there is a growing need to expand the olive varietal offer in olive groves for a number of reasons, such as resistance to water stress or water salinity, resistance to diseases, intensive farming, new areas of cultivation outside the Mediterranean basin or the search for specific quality characters in VOO. To do this, cultivars may be selected from the vast genetic pool that already exist or may be generated from the crossing of cultivars that are characterized by exhibiting a particular phenotype. Thus, in addition to traditional agronomic traits [18], the functional quality of VOO has been considered lately as a target of breeding programs that aim to obtain new olive cultivars [19,20,21]. With this in mind, the purpose of the present research was to conduct a study of the vitamin E content in olive. For this purpose, we have made use of the oils produced by a representative sample of olive (*Olea europaea* L.) cultivars from a World Olive Germplasm Collection (WOGC, IFAPA Alameda del Obispo). Also, the influence of the crop year climate and the degree of maturation of the olive fruit was studied. These data can be very useful to identify molecular markers for olive genotypes associated to high vitamin E in VOO, and for the selection of optimal parents in breeding programs in order to produce new cultivars with improved functional quality. In this sense, the vitamin E content was also studied in a segregated population of the ‘Picual’ and ‘Arbequina’ cultivars, which provides an idea of the suitability of the crosses to obtain new cultivars with an improved content of vitamin E in the oils.

## 2. Materials and Methods

### 2.1. Plant Material

VOO from two different gene pools of the olive species have been used in this study. On the one hand, 96 olive cultivars from the World Olive Germplasm Collection (WOGC, IFAPA Alameda del Obispo) located in Cordoba (Spain), which includes the 36 cultivars of a WOGC core collection that represents the genetic variability of the WOGC bank [22]. On the other hand, the segregating population of the cross of the cultivars ‘Picual’ and ‘Arbequina’, represented by 129 olive seedlings of the olive breeding program of Cordoba [23]. Trees from both gene pools were grown in the same edapho-climatic conditions, using drip irrigation and standard cultural practices, at the experimental orchards of IFAPA Alameda del Obispo in Cordoba.

To better compare the cultivars and seedlings when studying the genetic variability, fruits were picked by hand over the 2008–2017 crop years when they reached the turning stage, averaging a ripening index (RI) of 2.5 according to Beltrán et al. [24]. In the study of the effect of fruit ripening on the content of tocopherols, the fruits were also picked carefully by hand when the fruit color reached the yellowish-green, turning, and deep-purple stages, corresponding approximately to RIs of 1, 2.5, and 4, respectively [24].

### 2.2. Olive Oil Extraction

Olive fruits (2–3 kg) were used for olive oil extraction by means of an Abencor extractor system (Comercial Abengoa, S.A., Seville, Spain), which mimics the industrial process of VOO extraction at laboratory scale [25]. This system comprises a stainless-steel hammer mill operating at 3000 rpm equipped with a 5 mm sieve, a kneader operated for 30 min at 28 °C, and a basket centrifuge for the olive paste running at 3500 rpm for 1 min. The oils were decanted, paper-filtered, and stored under nitrogen atmosphere at −20 °C.

### 2.3. Analysis of Tocopherols

The analysis is based on the official IUPAC method [26] for tocopherols slightly modified. For this purpose, 50 mg of oil were dissolved in 1 mL of hexane containing α-tocopherol acetate (0.5 mg/mL) as internal standard, filtrated through 0.45 μm nylon and analyzed by high-performance liquid chromatography in a Beckman-Coulter system equipped with a Tracer LiChrosorb Si 60 column (250 × 4.6 mm, 5 μm) (Tecknokroma, Barcelona, Spain) and a Jasco FP-1520 fluorescence detector (JASCO Corporation, Tokyo, Japan). The elution was carried out at a flow rate of 1 mL/min, using as mobile phase hexane: isopropanol (99:1). The detection was performed at an excitation wavelength of 290 nm and an emission wavelength of 330 nm. For quantification, the internal standard and the response factors calculated for each of the tocopherols (α, β, and γ) was used. Data were expressed as mg of tocopherol per kg of oil.

## 3. Results and Discussion

So far, few studies have been conducted on the effect of genetics or the main agronomic factors—such as date of harvest, crop year, fertilization or irrigation—in the minor compound fraction of olive oil. In the present work, we have first studied the genetic factor responsible for the diversity of the content of tocopherols in the different olive cultivars. For this purpose, the content of tocopherols in the oils produced from two olive gene pools were assessed, cultivars from the WOGC collection, representative of the genetic variability of the olive species, and seedlings from a segregating progeny of the ‘Picual’ and ‘Arbequina’ cultivars. For guaranteeing the highest genetic diversity, the samples from the WOGC collection covers a high number of cultivars (96) from different Mediterranean countries, and it includes the 36 olive cultivars of a core collection [22].

**Figure 2:**
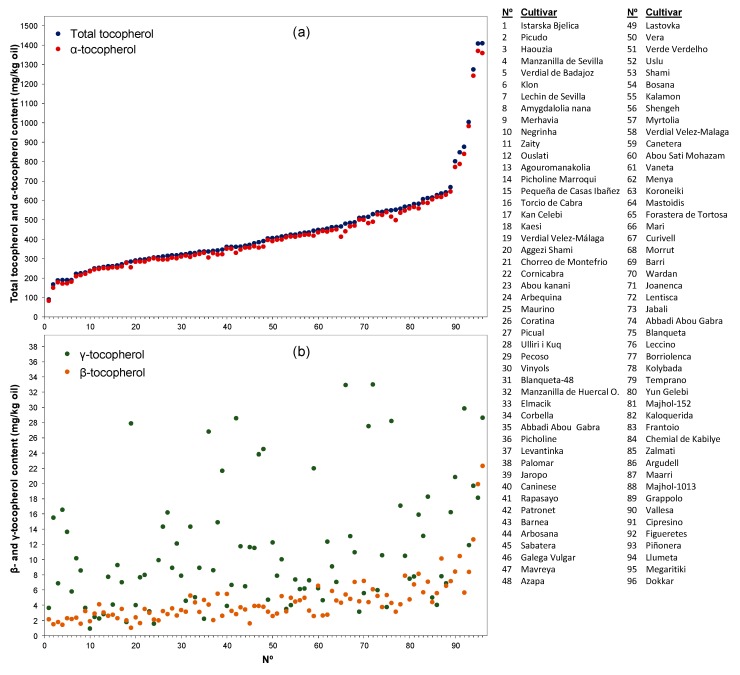
Content of tocopherols in the oils produced by 96 cultivars from the World Olive Germplasm Collection: (**a**) total tocopherol and α-tocopherol; (**b**) β- and γ–tocopherol. Olive fruits at turning stage were hand-picked over the crop years 2008–2017, processed for oil extraction in the same conditions and each sample analyzed twice for tocopherol content.

The genetic variability of the olive cultivars for tocopherol content in VOO is shown in Figure 2. The mean content of tocopherol was 444 mg/kg oil and the variability interval was 89–1410 mg/kg oil, whose ends correspond respectively to cultivar ‘Itarsca bjelica’ for the lowest value and cultivar ‘Dokkar’ for the highest. This content range is wider than that found previously by Beltrán et al. [12] when analyzing the content of tocopherols in the oil of 30 olive cultivars (84–510 mg/kg oil). Eight of these 30 cultivars have also been analyzed in this study displaying, in general, high coincidence with the levels of tocopherols found. Thus, oils from the cultivar ‘Istarska bjelica’ had the lowest content of tocopherols and the cultivar ‘Lentisca’ produced oils with high values of tocopherols (529 mg/kg oil). However, higher levels have been found in our survey for the oils from the cultivars ‘Frantoio’ (607 mg/kg oil) and ‘Leccino’ (549 mg/kg oil), which is in good agreement with the content reported by Špika et al. [27] for the latter cultivar. It has been observed that the cultivar ‘Frantoio’ shows a great variability by crop year, so it is not rare to find differences in the data provided by different studies carried out in different years for this cultivar. On the other hand, in the case of the cultivar ‘Mavreya’, the values found in different crop years are quite stable and are close to double to that reported by Beltrán et al. [12]. Cunha et al. [28] also carried out a study in 18 Portuguese cultivars and found that the content of tocopherols ranged from 93 to 260 mg/kg oil. Similarly, values of tocopherol content ranging 74–242 mg/kg in the oils produced from olive fruits at turning stage were found in a study conducted in five important Greek olive cultivars by Georgiadou et al. [14], which included four cultivars in common with our study (‘Kalamon’, ‘Mavreya, ‘Kaloquerida’, and ‘Koroneiki’). The contents of tocopherols in the present study were always higher than those found by Georgiadou et al. [14]. This variability observed for tocopherol content among olive cultivars is also found within the wild olive (oleaster). In this sense, Baccouri et al. [29] detected high genotype effect on the tocopherol content while evaluating the oil from seven different oleasters grown under similar agronomic, environmental, and technological conditions; the total tocopherols values ranged between 310 and 780 mg/kg oil.

**Figure 3:**
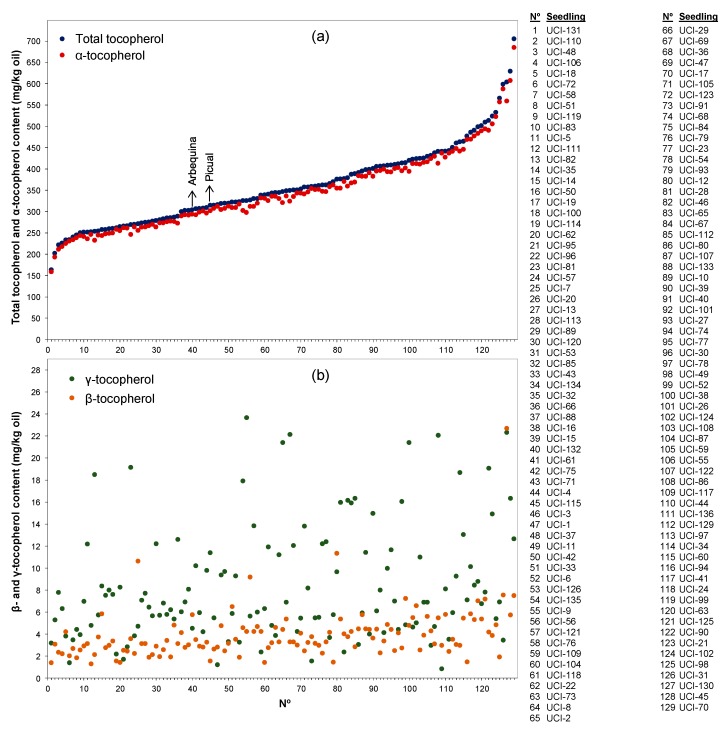
Content of tocopherols in the oils produced by one hundred twenty-nine seedlings from the segregating population of the cross of the cultivars ‘Picual’ and ‘Arbequina’: (**a**) total tocopherol and α-tocopherol; (**b**) β- and γ–tocopherol. The level of tocopherols of the genitors are marked in the (**a**) plot for comparison. Olive fruits at turning stage were hand-picked over the crop years 2008–2017, processed for oil extraction in the same conditions and each sample analyzed twice for tocopherol content.

The analysis of the content of tocopherols in the seedlings from a segregating progeny of the ‘Picual’ and ‘Arbequina’ cultivars also revealed a high level of variability (Figure 3), although lower than in the case of the cultivars of the WOGC collection. The mean content of tocopherol was 395 mg/kg oil and the variability interval was 289–706 mg/kg oil. This range of tocopherol content far exceeds the values found for the genitors of the progeny, ‘Picual’ (315 mg/kg oil) and ‘Arbequina’ (306 mg/kg oil), and suggest that any cross-combination could provide segregated populations with a wide range of variation for tocopherol content and be susceptible for use in breeding programs seeking cultivars with high vitamin E content. Data from this progeny showed to have a higher content of tocopherols than other breeding selections so far reported, such as those Tunisian seedlings coming from the cross of cultivars ‘Arbequina’ and ‘Chetoui’ (60–154 mg/kg oil) reported by Rjiba et al. [20].

As it has been traditionally considered, only the three forms of tocopherol-α, β, and γ—could be quantified in our study, and none of the other forms (δ-tocopherol and α, β, γ, and δ-tocotrienols) quantified by Georgiadou et al. [14] could be detected. The α-form was the major tocopherol, accounting for 96.16% on average of the total tocopherol content of the oils from the WOGC collection (Figure 2), and 96.54% from the oils of the segregating progeny (Figure 3). These results are similar to those found in the literature [12]. The immediate metabolic precursor of the α-form, γ-tocopherol (Figure 1), is the second tocopherol in content, reaching on average 2.81% among the cultivars of the WOGC collection and 2.36% for the seedlings of the crossing. Finally, the average level of β- tocopherol found was 1.03% and 1.10% for both olive samples, respectively.

**Figure 4:**
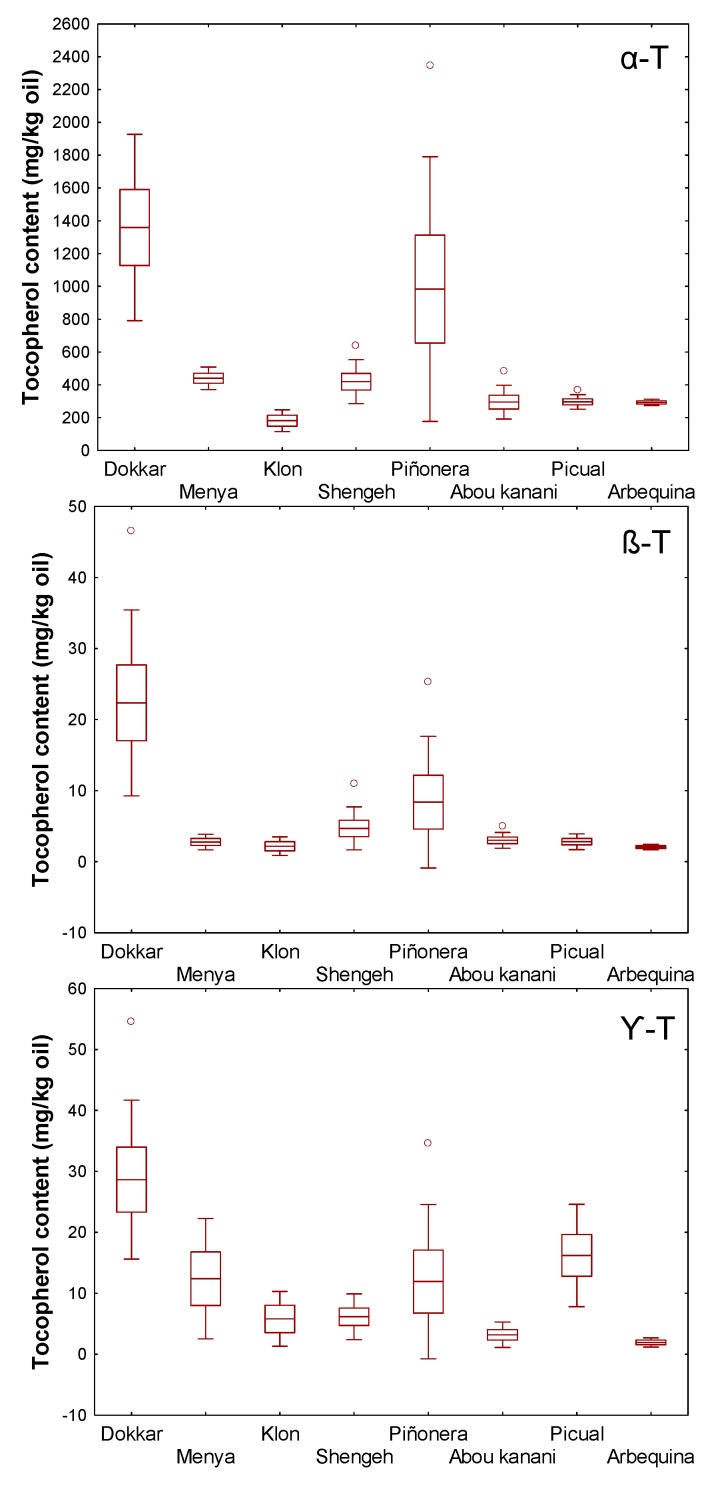
Variability across years for the contents (mg/kg oil) of the three forms of tocopherol in the oils from selected cultivars from WOGC: α-T, α-tocopherol; β-T, β-tocopherol; γ-T, γ-tocopherol. Horizontal inner lines in the boxes are mean values. The height in a box is equal to the standard error and the whiskers to standard deviation. The outliers (open dots) are indicated outside the whiskers.

The publication by Georgiadou et al. [14] reporting the detection of significant contents of tocotrienols in VOO is against the general assumption that dicotyledonous plants, like the olive tree, do not synthesize tocotrienols because their HPT enzymes has a strict substrate specificity for phytyl diphosphate (PDP). Apparently, this property is exclusive of monocotyledonous plants that contain a divergent form of the HPT—that is, HGGT—which shows substrate specificity for geranylgeranyl diphosphate (GGDP) in preference to PDP (Figure 1) [30]. In this sense, we have recently generated a series of cDNA libraries from selected cultivars of the WOGC collection whose oils showed considerable differences in the contents of tocopherols (shown in Figure 4) to study their transcriptomes. On the basis of the sequence annotation by three independent annotation programs and further manual screening from the NCBI database, we could not find any gene corresponding to HGGT in olive. In contrast, Georgiodou et al. [13,14] found a homolog of an expressed sequence tag (EST) of HGGT in the OLEA EST database obtained by Alagna et al. [31]. According to what the NCBI database shows, this EST actually corresponds to a fragment of an HPT gene. In this sense, we have only identified six likely transcripts encoding HPT enzymes from our sequencing data, although only one of them displays relevant expression levels (unpublished results). The content of tocotrienols in the olive oil reported by Georgiodou et al. [14] could be a consequence of a residual activity with GGDP of one of the HPT enzymes or the enzyme encoded by the single gene *homogentisate solanesyltransferase* that we have also found in our sequencing data from olive. This enzyme uses as main substrate solanesyl diphosphate, an isoprenoid residue structurally similar to the GGDP but composed of 45 carbons, which is involved in the plastoquinone-9 biosynthesis.

**Figure 5:**
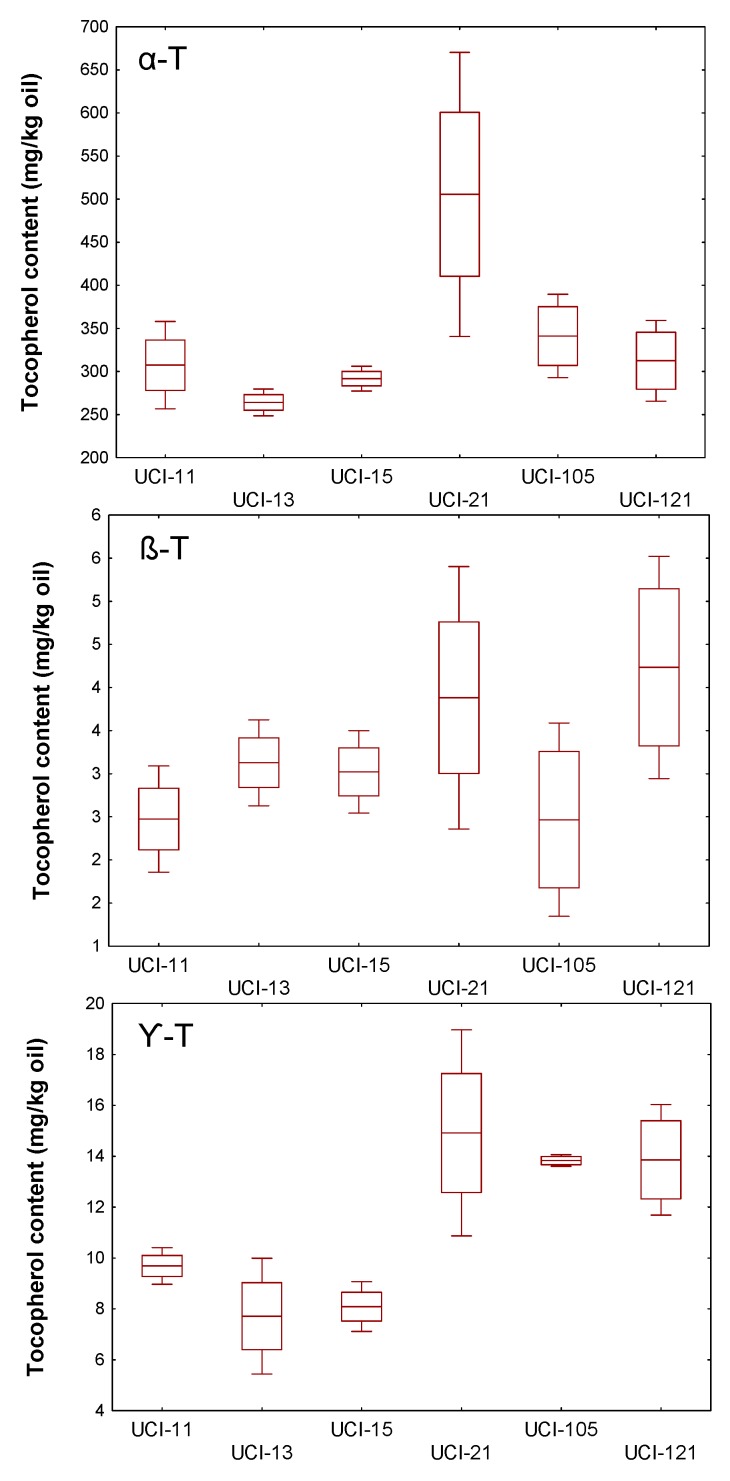
Variability across years for the contents (mg/kg oil) of the three forms of tocopherol in the oils from selected seedlings from the cross of ‘Picual’ and ‘Arbequina’: α-T, α-tocopherol; β-T, β-tocopherol; γ-T, γ-tocopherol. Horizontal inner lines in the boxes are mean values. The height in a box is equal to the standard error and the whiskers to standard deviation.

In order to investigate how the variation of the climatic conditions across years affects the content of tocopherols, as described in Materials and Methods, the trees of the WOGC collection and the segregating progeny were grown under the same agronomic (i.e., fertilization and irrigation) and edapho-climatic conditions, the fruits were carefully hand-picked at the same stage of ripeness (turning stage, around RI = 2.5), and the oil extracted through the same processing system. Thus, the content of tocopherol in the olive oils would only be affected by its crop year. For this purpose, six cultivars from the WOGC collection displaying important differences in terms of tocopherol content were selected, and the content of these compounds monitored at least in four different crop years in the 2008–2017 period (Figure 4); the cultivars ‘Dokkar’ and ‘Piñonera’ as representative of high tocopherol cultivars (>400 mg/kg oil), ‘Menya’ and ‘Shengeh’ as medium content cultivars (300–400 mg/kg oil), and ‘Klon′ and ‘Abou kanani’ as representative of low-level tocopherol cultivars (< 300mg/kg oil). In addition, the study has been extended to cultivars ‘Picual’ and ‘Arbequina’ given their commercial significance since they are two of the most cultivated olive cultivars in the world, and because they are also the parents of the ‘Picual’ x ‘Arbequina’ cross (Figure 4). Figure 5 shows the data obtained from the study of six seedlings from the progeny ’Picual’ x ‘Arbequina’ exhibiting marked differences in the content of tocopherols; being UCI-21 and UCI-105 as representative of high-level tocopherol seedlings (>400 mg/kg oil), UCI-11 and UCI-121 as medium content seedlings (300–400 mg/kg oil), and UCI-13 and UCI-15 as representative of olive seedlings displaying low contents of tocopherol (<300 mg/kg oil). In general, the cultivars of the WOGC collection and seedlings of the cross displaying high levels of tocopherols in the oil also presented greater variability by crop year, and this occurs for all α-, β-, and γ-forms (Figure 4; Figure 5). Thus, ‘Dokkar’ and ‘Piñonera’ cultivars have coefficients of variation (CV) of 42% and 82%, respectively, both for α-tocopherol and for the sum of the content of all forms of tocopherols. The same occurs in the case of the crossing seedlings, where the UCI-21 reaches a CV of 160%. Even so, cultivars from the WOGC collection with low tocopherol levels also have considerable CV values, 35% for ‘Klon’ and ‘Abou kanani’ cultivars. By contrast, the seedlings selected among the progeny of the cross characterized for having low tocopherol contents, UCI-13 and UCI-15, seem to behave more homogeneously in the different crop years, with their CVs not exceeding 6%. In general, the CV of the studied seedlings are in the range of 6–16%. These values are similar to those showed by the two parents of the cross (‘Picual’ and ‘Arbequina’) with values of 16% and 6%, respectively. The only exception is the seedling UCI-21, which is characterized by having high levels of tocopherol. As expected, the variability by the crop year found are very similar for the α-form and for the sum of all the forms of the tocopherols (Figure 4 and Figure 5). For the rest of the forms (β- and γ-tocopherols), the variability presents a pattern similar to that of the α-form, although in general with a slightly higher value. The results found regarding the effect of the climatic conditions of the year on the content of tocopherols in VOO are in agreement with Georgiadou et al. [13], who found a CV of 41% for cultivar ‘Koroneiki’ at the ripening stage in a study of three consecutive crop years. Similar conclusions were reached by Beltrán et al. [12] for cultivars ‘Picual’, ‘Hojiblanca’, and ‘Frantoio’, but not with what was collected in a previously published review by Uceda et al. [32], which reported a low level of influence of the crop year. Significant effect of genotype and crop year was also found in other study including breeding selections from the same parentage than this work [33]. 

**Figure 6:**
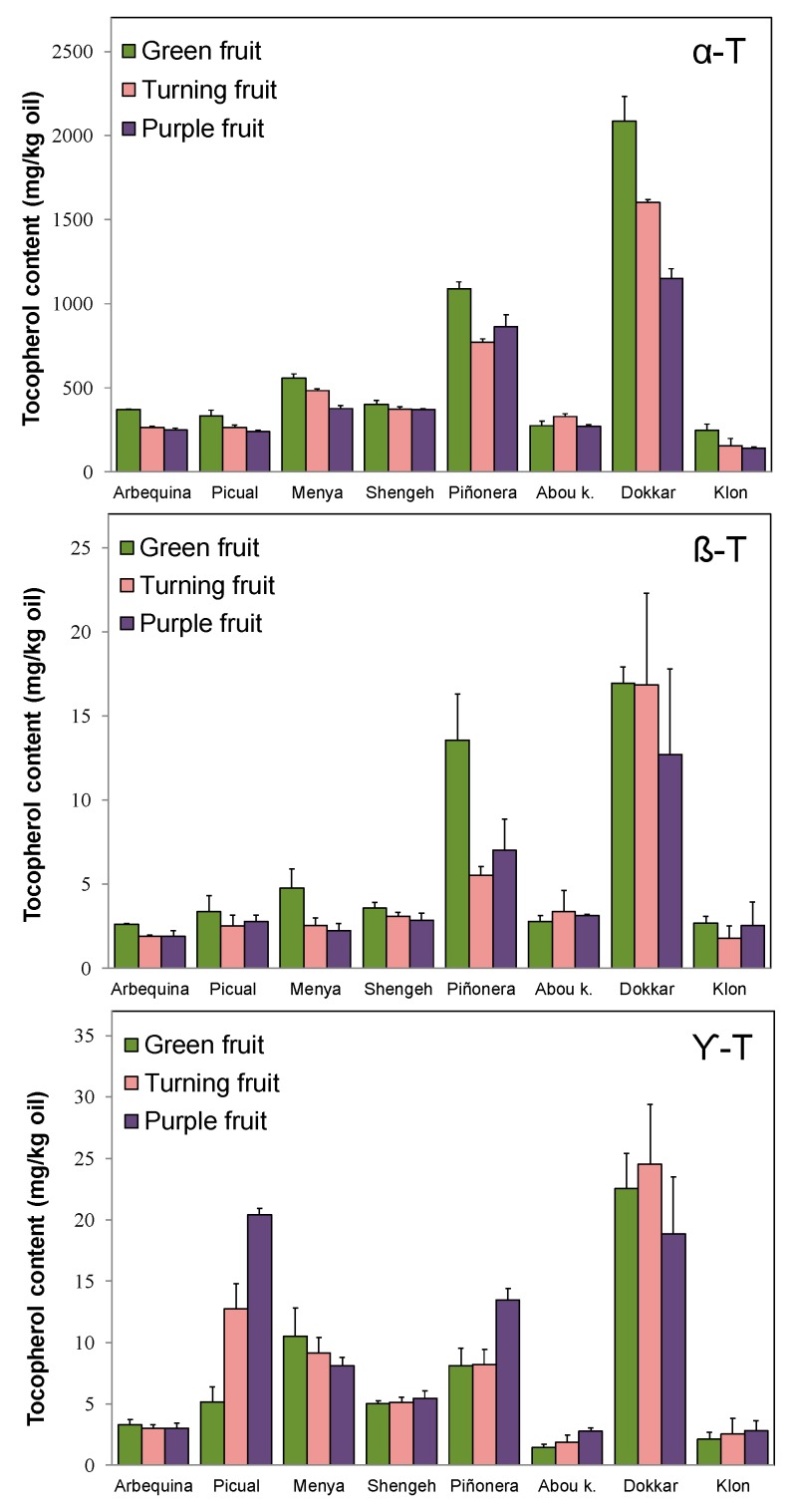
Changes in the contents (mg/kg oil) of the three forms of tocopherol in the oils from selected cultivars from the World Olive Germplasm Collection along the olive fruit ripening (average contents and standard deviations from at least three different crop years): α-T, α-tocopherol; β-T, β-tocopherol; γ-T, γ-tocopherol.

Figure 6 shows the average content of each tocopherol form found along three different ripening stages of the olive fruit. The content of these compounds has been monitored in the six selected cultivars of the WOGC collection, in addition to the more commercial ‘Picual’ and ‘Arbequina’ cultivars, throughout different crop years. Data suggest that the evolution of the tocopherol content throughout the maturation of the olive fruit is a fairly conserved process in each cultivar, dependent, therefore, on its particular physiology. In general terms, a decrease in tocopherol content is observed in most cultivars, except for cultivars ‘Shengeh’ and ‘Abou kanani’ whose levels of tocopherols barely changes during the fruit ripening process, and cultivar ‘Piñonera’ that displays almost every crop year a characteristic increase in tocopherol content in advanced stages of fruit ripening (purple fruit), of which the figure corresponding to α-tocopherol in Figure 6 is very representative. This general tendency to decrease the content of tocopherols during the ripening of the fruit was already observed in cultivars ‘Hojiblanca’ and ‘Picual’ by Gutiérrez et al. [11], and later corroborated by Beltrán et al. [12] in the same cultivars. However, the results found for the cultivar ‘Frantoio’ are discordant. While Ranalli et al. [10] found that levels of tocopherols increased during fruit ripening to decrease as the fruit enters the senescence stage, Beltrán et al. [12] found a steady decline during the ripening process, as we have found in this study for most of the cultivars under study.

Particularizing in each form of tocopherol, the β-form follows a pattern very similar to that of the α-form during fruit ripening. However, the content of the γ-form keeps a different evolution. In most of the cultivars studied, there is an increase in the γ-tocopherol content throughout the ripening of the fruit. This singularity was already noticed by Ranalli et al. [10] in cultivars ‘Frantoio’ and ‘Leccino’, whereas Beltrán et al. [12] observed the same in cultivars ‘Hojiblanca’ and ‘Picual’, suggesting that the γ-tocopherol increase is consequence of the breakdown of chlorophylls in the olive fruit as it ripens. Coinciding with the results already published, the cultivar ‘Picual’ is characterized by a sustained increase of γ-tocopherol content during the olive ripening process (Figure 6). It is striking that the other cultivar of commercial relevance, ‘Arbequina’, despite presenting tocopherol levels and a fruit ripening trend very similar to those of cultivar ‘Picual’, however it does not show this particular pattern of γ-tocopherol accumulation.

## 4. Conclusions

A high degree of variability for the content of tocopherol in VOO is found in the olive species. Findings indicated that tocopherol content and composition were highly dependent on cultivar and, to a lesser extent, on the year’s climate and the fruit ripening stage. The possibility of interaction between both variables and that derived of the conditions of the olive tree culture could not be excluded. In general, the cultivars from the WOGC collection and the seedlings of the cross that display higher levels of tocopherols in the oil present a greater variability due to crop year than the rest of the genotypes. This has been observed for all forms of tocopherols (α-, β-, and γ-forms). The present study has also shown that is possible to obtain a high degree of variability for the content of tocopherols in VOO with just a single cross of olive cultivars. This variability greatly exceeds the parental contents, both above and below these contents. Therefore, in breeding works, aiming at obtaining new olive cultivars with improved content of vitamin E, it seems more effective to consider a greater number of individuals within the same cross that use different crosses with fewer individuals [23,34]. This information could be very useful for further molecular-assisted screening and breeding of olive. In this sense, it is of great interest to search for molecular markers that allow the identification at the seedling stage of genotypes that produce oils with higher levels of tocopherol to avoid the long development time necessary for the breeding programs of woody plants such as the olive.
